# ECMIS: computational approach for the identification of hotspots at protein-protein interfaces

**DOI:** 10.1186/1471-2105-15-303

**Published:** 2014-09-16

**Authors:** Prashant Shingate, Malini Manoharan, Anshul Sukhwal, Ramanathan Sowdhamini

**Affiliations:** National Centre for Biological Sciences (TIFR), GKVK Campus, Bellary Road, Bangalore, 560065 India; Manipal University, Madhav Nagar, Manipal, 576104 Karnataka India

**Keywords:** Protein-protein interactions, Domain interfaces, Protein superfamily, Drug design

## Abstract

**Background:**

Various methods have been developed to computationally predict hotspot residues at novel protein-protein interfaces. However, there are various challenges in obtaining accurate prediction. We have developed a novel method which uses different aspects of protein structure and sequence space at residue level to highlight interface residues crucial for the protein-protein complex formation.

**Results:**

ECMIS (Energetic Conservation Mass Index and Spatial Clustering) algorithm was able to outperform existing hotspot identification methods. It was able to achieve around 80% accuracy with incredible increase in sensitivity and outperforms other existing methods. This method is even sensitive towards the hotspot residues contributing only small-scale hydrophobic interactions.

**Conclusion:**

Combination of diverse features of the protein *viz.* energy contribution, extent of conservation, location and surrounding environment, along with optimized weightage for each feature, was the key for the success of the algorithm. The academic version of the algorithm is available at http://caps.ncbs.res.in/download/ECMIS/ECMIS.zip.

**Electronic supplementary material:**

The online version of this article (doi:10.1186/1471-2105-15-303) contains supplementary material, which is available to authorized users.

## Background

Protein-protein interactions are vital for many cellular processes like signal transduction, DNA replication, cellular motion, and transport of molecules from one cell to another. Free energy is an important criterion for protein-protein binding and hence for better understanding of protein-protein interactions. The contribution of various interface residues towards free energy of binding is not uniform [1, 2] and the ones which are energetically more important are known as hotspots. Hotspot residues are defined as those which bring changes in the binding free energy by more than 2 kcal/mol, when mutated to alanine [3]. These residues are generally seen to exist in clusters known as ‘hot regions’ [4]. Such hotspot regions provide stability to the protein complexes and also attribute specificity to their binding sites [1, 2, 5]. Alanine Scanning Energetic Database (ASEdb) [6] contains a list of hotspots from some selected proteins where they were mutated to alanine and changes in free energy of binding were recorded. Binding Interface Database (BID) [7] is another database which collects information on hotspot residues from literature studies.

The amino acid compositions of hotspot and non-hotspot residues are slightly different [2]. Residues like Tyr, Arg and Trp have higher tendency to be a hotspot residue, because of their size and conformation [2], while other residues like Leu, Thr, Ser and Val are less prevalent [2, 5]. Asp and Asn have been observed to contribute critically to hotspots, more frequently than Glu and Gln. This might be attributed to the differences in their side chain conformational entropy [2, 5]. Some studies have also indicated that hotspot residues are more conserved than non-hotspot residues [8, 9]. Hotspot residues have been observed to be surrounded by residues which are moderately conserved [4] and play a part in occluding the bulk solvent from the hotspots [10, 11]. This occlusion of hotspot residues from the bulk solvent is found to be the major reason for their highly efficient interactions with other residues at the interface. Residues at protein-protein interfaces have been studied for their conserved nature [12–15] and those residues which are structurally and functionally [16] important tend to remain evolutionarily conserved or mutate at a slower pace as compared to the rest of the protein. Further studies maintain that conserved residues remain highly buried in the protein surface [17, 18]. Hotspot residues have been found to correlate well with the conserved residues at the interfaces [17, 19] and found to be buried and tightly packed within the interface [4].

Since identification of hotspot residues by experimental methods like alanine scanning mutagenesis [20], alanine shaving [21] and residue grafting [21] is both expensive and time consuming, their characteristics have been greatly exploited by a number of computational methods which can predict and identify these hotspot residues from the interface ones. In recent years, several computational methods have been developed which uses one or more characteristics of hotspots, as described above, to identify and successfully predict them from the set of interface residues.

ROBETTA [22] uses a simple physical model which measures changes in binding energy of the complex when a residue is mutated to Alanine. It was applied on a large dataset obtained from ProTherm and ASEdb. FOLDEF [23] uses atomic descriptors of protein structures and various energy terms weighted based on empirical data as obtained from experimental data. It was trained on 339 mutations as obtained from 9 different proteins and the various parameters were optimised. This was then tested on 667 mutations from 82 protein-protein complexes.

There are several machine learning methods available *e.g.* KFC [24] uses a machine learning approach to characterize its local structural environment and then compare it with the environments of experimentally determined hotspots. If the environment of the interface residue resembles the experimentally determined hotspots, then it is predicted as a hotspot. The method was trained on 249 experimentally characterised mutations from 16 non-redundant protein-protein complexes and tested on an independent test dataset of 112 mutations. MINERVA [25] uses a support vector machine (SVM) based approach, wherein various structure, sequence and molecular interaction parameters are used to predict hotspots. HotPoint [26] is based on an empirical model which uses features like solvent occlusion and knowledge-based pair potential of residues to predict hotspots. KFC2 [27] uses a SVM-based approach, wherein solvent accessibility and local plasticity of the residues are used as features to predict hotspots. Most of these methods are trained on a subset of Alanine Scanning Energetic Database (ASEdb) and tested independently on a dataset obtained from Binding Interface Database (BID).

Other methods use features like solvent accessibility [28–30], atomic contacts [31], restricted mobility [17], location in the interaction patch [4], structural conservation [32], sequence conservation [29, 33–35], sequence environment and evolutionary profile [36], and pattern mining [37] to identify hotspot residues. Although these methods alone provide reasonable information about the hotspot residues, it has been observed that these cannot be used for the prediction/identification of hotspot residues with high accuracy [38]. Some of the methods employ energy functions [23, 39] while others use molecular dynamics simulations [40]. Various machine learning approaches [3, 24, 25, 41–43], based on geometry and biochemical features of residue-residue contacts across binding interfaces, have also been developed to identify hotspot residues. Simple empirical method based on residue-residue pairwise potentials and surface accessibility [26], and a different method which uses protein docking tools [44], have also been developed which identifies hotspot residues with fairly good accuracy. Robetta [22] was one of the first methods developed to identify hotspot residues, which accounted for energies of packing interactions, hydrogen bonds and solvation [45]. Molecular dynamics (MD) simulations have also been used and found to provide good predictive results for hotspot prediction [46]. However, MD simulations cannot be used for large scale prediction of hotspot residues, since they are computationally very intensive.

In this paper, we present a new method “Energetic Conservation Mass Index and Spatial Clustering” (ECMIS) which uses a combination of interface energetic (non-covalent interactions like hydrogen bonds, Van der Waals and electrostatics), residue conservation, mass-index and spatial clustering to predict hotspot residues with higher accuracy than any of the other methods available. ECMIS considers most essential and carefully selected distinguishing features of hotspot residues, along with optimum weightage, to calculate combined score for each position. Hence, ECMIS was able to achieve high sensitivity compared to other methods.

## Method

### Dataset

- Training set
  A dataset of 316 alanine-mutated interface residues (Additional file 1) derived from 19 protein complexes was taken from ASEdb [6]. Residues in the dataset corresponding to a binding free energy equal to or higher than 2.0 kcal/mol were alone considered as a hotspot residues. The interface residues with binding free energy less than 0.4 kcal/mol were considered as non-hotspot residues, as described by Tuncbag *et al*. [30] and Xia et al. [47]. Other interface residues with binding free energy between 0.4 and 2.0 kcal/mol were excluded from the training set, in order to better discriminate between hotspots and non-hotspots. The final training dataset comprised of 78 hotspot residues and 119 non-hotspot residues. The program was optimized based on the prediction accuracy of the hotspots in this dataset with varying parameters. The entry 1DN2 has been removed from the dataset, since the protein is complexed with an artificial peptide and therefore the conservation based scores cannot be applied.
- Test set
  An independent test set from the BID database [7] (Additional file 2) was used to further assess the performance of our proposed method. The residues in BID database, are categorized as ‘strong’, ‘intermediate’, ‘weak’ or ‘insignificant’ based on the effect of the mutation. The residues labeled as ‘strong’ were considered as true hotspot and the other residues are considered as non-hotspots. As a result, the test set contained 125 alanine-mutated interface residues in 18 protein complexes with 38 hotspots and 87 non-hotspots.

### Dataset for calculation of energy ranges

PPCheck is a program used for calculating energies at protein-protein interfaces and the energy ranges have been benchmarked earlier on 246 complexes (Sukhwal and Sowdhamini, 2013) [48]. These PDB complexes were obtained at a resolution of 2.5 Å or better, constituting 270 protein-protein interfaces (water excluded from interface) in order to define the energy ranges for the three energy components viz. electrostatic-energy, Van der Waals interaction energy and hydrogen bond energy. This benchmarking dataset had included homodimers, heterodimers, transient and permanent complexes, antigen-antibody complexes, *etc.* [48].

### Energy scoring scheme

The energy contribution per residue was examined, as reported in PPCheck. Energy values from PPCheck involve three energy components viz. electrostatics, Van der Waals interactions and hydrogen bond energy. Further scripting was done to extract energy values in a residue-centric manner. Energy component for each residue was weighted to calculate final energy score. These weights were decided based on the application and performance on training dataset (Additional files 3 and 4).



Where  = Total binding energy contributed by i^th^ residue

 = Van der Waals interaction energy contributed by i^th^ residue

 = Electrostatic interaction energy contributed by i^th^ residue

 = Hydrogen bond energy contributed by i^th^ residue

*w*_*VW*_ = Optimized weight for Van der Waals interaction energy

*w*_*ES*_ = Optimized weight for electrostatic interaction energy

*w*_*HB*_ = Optimized weight for hydrogen bond energy

Energy per residue was then normalized with respect to the volume of the residue to reduce bias due to size of the interacting residues.


Where  = Volume normalized total interaction energy contributed by i^th^ residue

*V*_*i*_ = Volume of i^th^ residue

These volume-normalized scores were further normalized using observed energy ranges of all component energies.


Where *E*^*i*^ = Final binding energy of i^th^ residue normalized between 0–1

*ne*_max_ = Maximum volume normalized interaction energy observed in large dataset of different protein complexes

### Conservation score

Along with energy score, the extent of evolutionary conservation for each residue was calculated. First, homologues were searched using PSI-BLAST [49] tool and homologues having blast identity more than 30% were chosen. Further, redundancy amongst homologous sequences was addressed by applying a filter at 80% sequence identity by using CD-HIT [50]. The threshold of 80% was found as the best value to remove highly similar sequences as well as maintaining optimum number of homologues required for accurate multiple sequence alignment (Additional file 5). This was performed to calculate the conservation score without any bias due to closely related sequences. All the homologues, along with query, were aligned using ClustalW [51] software. Each position was then individually checked for conservation and assigned a conservation score as per Johnson and Overington matrix [52]. This matrix was derived using structure-based sequence alignment of homologous protein families. A similar approach was used earlier in Smotif [53] algorithm, which proved to be very efficient in finding structural motifs.



Where *c*^*i*^ = Normalized total conservation score of i^th^ position

a = Residue type present in homologous sequence at i^th^ position in multiple sequence alignment

b = Residue type present in another homologous sequence at i^th^ position in multiple sequence alignment

*S*^*ab*^ = Amino acid substitution score residue type “a” substituted by residue type “b” from Birkbeck matrix

n = Total number of homologues present in the multiple sequence alignment

All scores were further normalized by 100 (maximum possible score i.e. cysteine-cysteine substitution score in Johnson and Overington matrix [54]).


*C*^*i*^= Final conservation score normalized between 0–1

### Mass index score

For each interface residue, sum of mass of interacting residues were calculated as Mass Index score.


Where *mi*^*i*^ = mass index of i^th^ residue

*m*^*i*^ = mass of i^th^ residue

*m*^*j*^ = mass of j^th^ residue

j = j^th^ residue interacting with i^th^ residue

n = Total number of residues interacting with i^th^ residue

*mi*_max_ = Maximum mass index in large dataset of different protein complexes

*MI*^*i*^ = mass index of i^th^ residue normalized between 0–1

### Spatial clustering

Hotspot residues could cluster spatially and forms hot-regions [4]. This fact was used to further enhance score of those hotspot residues which forms very efficient and conserved binding patch. To achieve this, average of energy and conservation scores was referred. If this average score for any residue exceeds 0.5, then its score will be further enhanced with respect number of other hotspot residues within 7 Å spatial proximity.


Where *sc*^*i*^ = Spatial cluster score for i^th^ residue

 = Number of residues present within same protomer within 7 Å distance of i^th^ residue

 = Number of residues present within interacting protomer within 7 Å distance of i^th^ residue

*sc*_max_ = Maximum spatial cluster score observed in large dataset of different protein complexes

*SC*^*i*^= Final spatial cluster score for i^th^ residue normalized between 0–1

### Final score

Final score was calculated by combining energy score, conservation score and spatial clustering score. Each subscore was weighted according to their importance in identifying hotspots. These weights were applied along with threshold score (decided using ROC plots) to decide hotspot criteria and were empirically optimized based on the minimization of residual error in the prediction using training dataset. Here, the reduction in the number of false positives and false negatives were considered as optimization function.


Where *f*^*i*^ = Final combined score of i^th^ residue

*w*_*E*_ = Optimized weight for energy score

*w*_*C*_ = Optimized weight for conservation score

*w*_*SC*_ = Optimized weight for spatial clustering score

*w*_*MI*_ = Optimized weight for mass index

*f*_max_ = Maximum combined score observed in data

*F*^*i*^= Final combined score of i^th^ residue normalized between 0–1

### Performance evaluation

In order to assess the performance of classification methods, commonly used measures such as prediction accuracy (ACC), sensitivity (SE), precision (PR), specificity (SP) and Mathews Correlation Coefficient (MCC) were used. These measurements are defined as


Where TP, FP, TN and FN represent true positive (correctly predicted hotspot residue), false positive (non-hotspot residue incorrectly predicted as hotspot), true negative (correctly predicted non-hotspot residue) and false negative (hotspot residue incorrectly predicted as non-hotspot), respectively.

## Results and discussions

### Optimization of the parameters of ECMIS

All the parameters *viz.* weights for each type of component energy (*vizN* electrostatic energy, Van der Waals energy and hydrogen bonding), weights for each type of score and threshold hotspot score were optimized empirically. The maximum accuracy was achieved using optimized values of these parameters, mentioned in Table 1).

**Table 1:** **Empirically optimized set of parameters**

Parameters	Value
*w*_*ES*_	1
*w*_*HB*_	9
*w*_*VW*_	1.4
*w*_*E*_	0.3
*w*_*C*_	0.9
*w*_*MI*_	0.4
*w*_*SC*_	0.4

**Figure 1:**
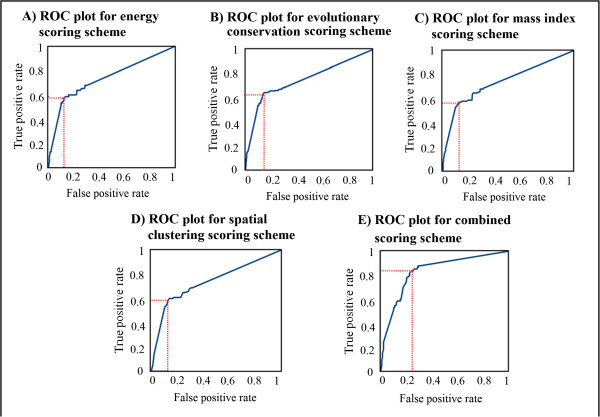
**ROC plots for: A) Energy score, B) Conservation score, C) Mass index score, D) Spatial clustering score and E) Combined score.** The red line corresponds to best threshold score with optimum trade-off between true positive rate and false positive rate.

The best value for the discriminative threshold scores (Table 2) for all scoring schemes were decided after consulting respective ROC plots (Figure 1). ROC – Receiver operating characteristics plot is one of the methods which can be used to decide a threshold value for the given parameter at which an optimum performance for the algorithm can be achieved. ROC curve graphically represents gain in true positive rate with the expense of false positive rate. The point after which increase in true positive rate is smaller compared to increase in false positive rate selected as a threshold value shown in red (Figure 1; Additional file 6).

**Table 2:** **Threshold scores for each component scoring scheme**

Scoring scheme	Threshold score
Energy score	0.58
Conservation score	0.68
Mass index score	0.50
Spatial clustering score	0.54
Combined score	0.80

While optimizing weights for individual component energy, it was observed that hydrogen-bond energy was always over-represented in case of threonine and serine. Hence their mass-index values (MI_Ser_ > 0.5, MI_Thr_ > 0.5) was considered as additional criteria to reduce false positive in case of serine or threonine residues. In contrast tryptophane and phenylalanine mostly contributed in Van der Waals energy which is further normalized by their volume. Compared to other types of energies, magnitude of Van der Waals energy was very small while volume of phenylalanine and tryptophan was high compared to other amino acids. Therefore these residues always get smaller score irrespective of their importance in protein-protein complex formation. To overcome this problem again mass index score (MI_Trp_ > 0.5, MI_Phe_ > 0.5) were consulted to improve scores of true positive tryptophan and phenylalanine residues.

### Normalization of scores

**Figure 2:**
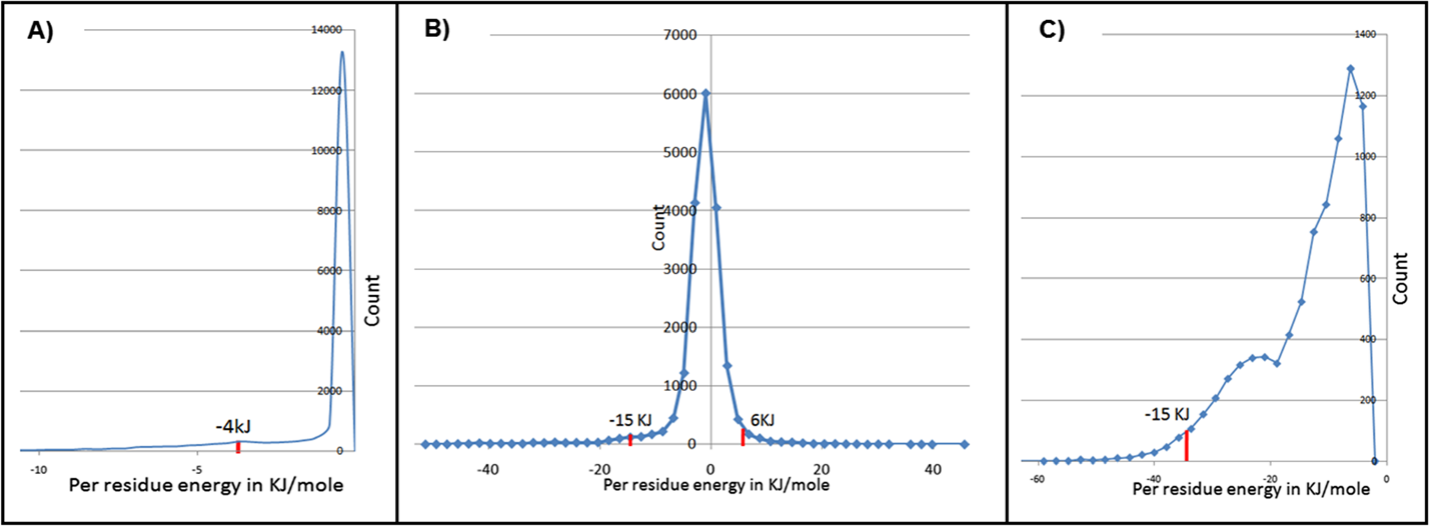
**Energy ranges for: A) Van der Waals interactions B) Electrostatic interactions C) Hydrogen bonding energy.**

In order to compare scores obtained for one protein complex with another protein complex all scores were normalized using maximum value observed for each parameter in dataset of diverse protein complexes (Figure 2). These ranges were decided after considering 95% of the data and extreme 5% were ignored.

### Prediction of the independent test set

The optimized parameters were used for the identification of hotspot residues in the independent test dataset from the BID database. Our algorithm was able to achieve an accuracy of approximately 80% on the test dataset for optimized set of weights (Table 1).

### Comparison of the method with other methods

ECMIS was compared with various other methods available for the identification of hotspot residues Robetta [22] and FOLDEF [23], decision tree methods such as KFC [24] and three recently published methods MINERVA [25], HotPoint [26], KFC2 [27] and random forest based methods [43]. An independent test set from the BID database with 125 alanine-mutated interface residues in 18 protein complexes with 38 hotspots and 87 non-hotspots was used. The performance of these methods on the test set is listed in Table 3. ECMIS performs better than the currently available methods with an accuracy of 80% and MCC of 0.524.

**Table 3:** **Comparison of ECMIS with other prediction methods**

Method	PR (%)	SE (%)	SP (%)	ACC (%)	MCC
ECMIS	68%	66%	87%	80%	0.524
RF	70.8	44.7	92.0	77.6	0.429
MINERVA	65.4	44.7	89.7	76.2	0.390
KFC2	58.1	47.4	85.1	73.6	0.345
HotPoint	49.0	63.2	71.3	68.8	0.324
Robetta	52.0	34.2	86.2	70.4	0.235
KFC	48.0	31.6	85.1	68.8	0.191
FOLDEF	47.6	26.3	87.4	68.8	0.168

### Case studies

**Figure 3:**
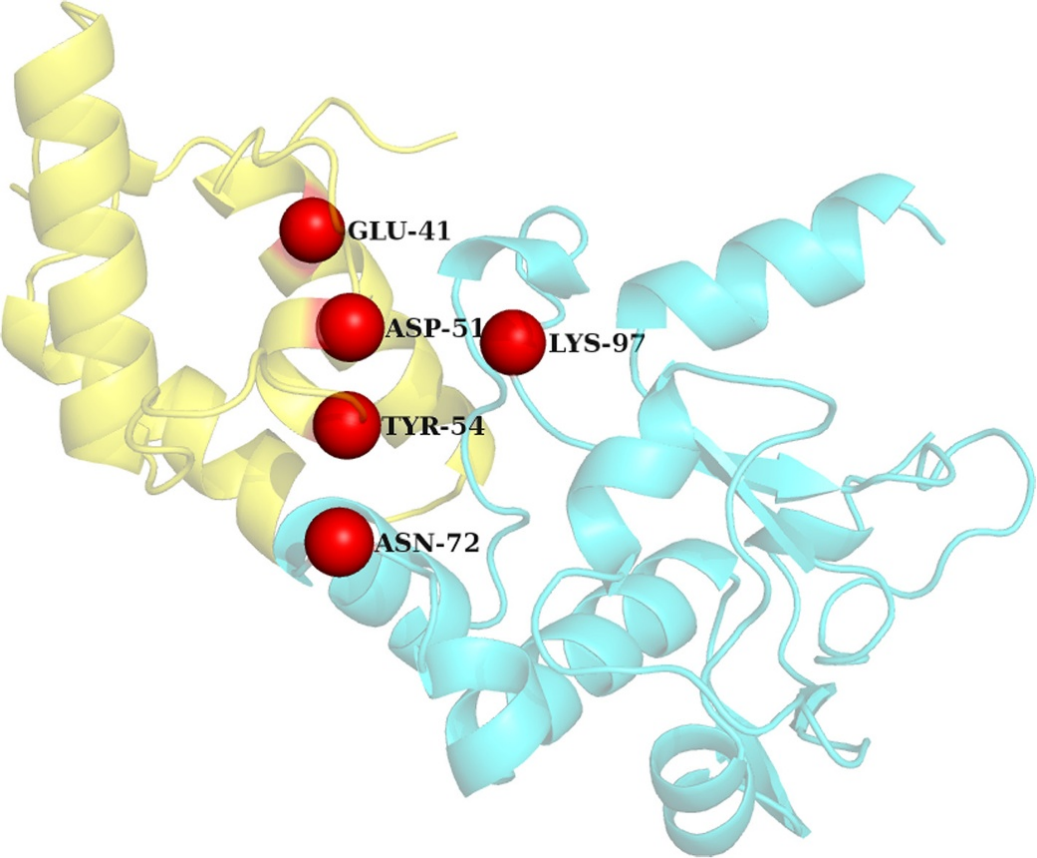
**Interaction between Subtilisin BPN’ precursor (blue) and Chymotrypsin inhibitor 2 (green) [PDB ID: 2VLQ]: The true hotspot residues identified by ECMIS in Subtilisin BPN’ precursor are represented in red color while the true hotspot residues identified in Chymotrypsin inhibitor 2 are represented in orange color.**

**Figure 4:**
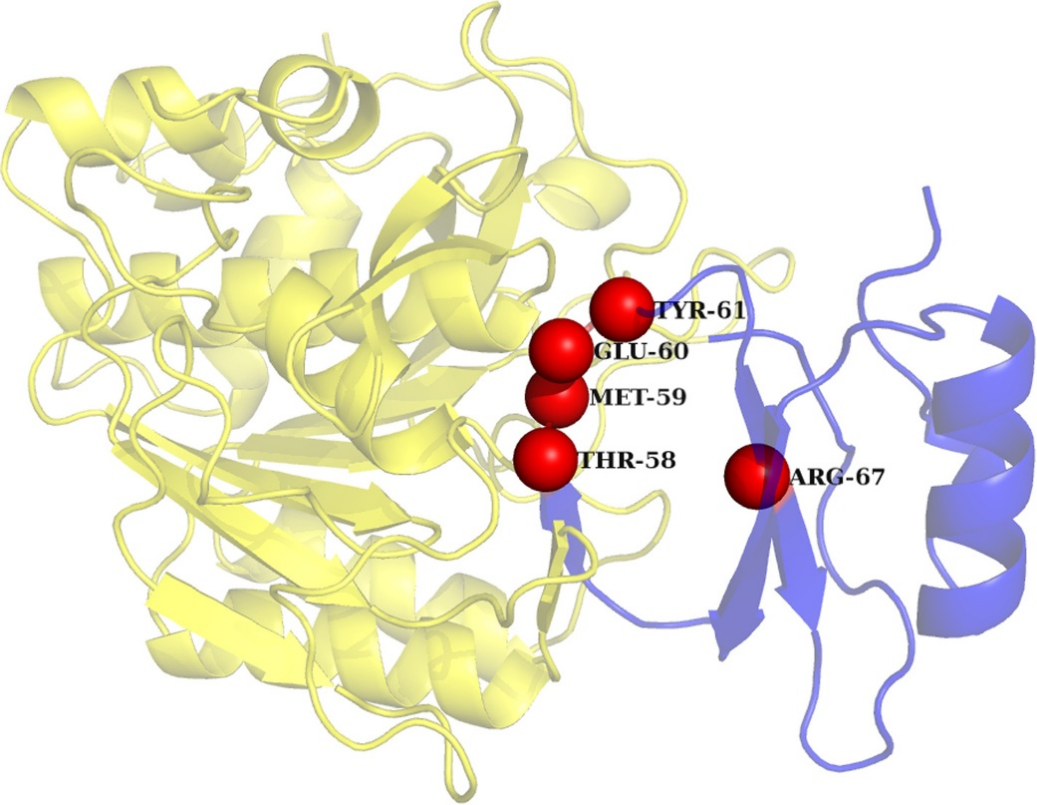
**Interaction between Colicin endonuclease (green) and Im9 (blue) [PDB ID: 1TM1]: The true hotspot residues identified in Colicin endonuclease by ECMIS are represented as red spheres.**

- Colicin endonuclease-Im9 complex
  A random protein from the PDB was chosen to demonstrate the performance of our hotspot identification algorithm. Colicin endonucleases (DNases) are bound and inactivated by immunity (Im) proteins. A number of hotspot residues have been identified by mutagenesis which affects the binding of the DNase-1 m9 complex ((PDBID: 2VLQ). It has been shown that the mutation of three Im9 residues of helix III Asp51, Tyr54 and Tyr55 to alanine generates change in the energy values of ΔΔ*G* > 5 kcal/mol) [54]. In the case of E9 DNase three important residues (Asn75, Phe86 and Lys97) form a central belt on the surface of the enzyme that comprises the hotspot. Additionally the salt-bridge between Glu41 of Im9 with Lys97 of the E9 DNase has been shown to be a specificity contact in this complex [55]. Arg54 and Asn72 of E9 DNase have also been found to effect the binding of DNase to Im9 protein. It was observed that ECMIS was able to pick 2 out of the 5 hotspot residues in Dnase (Lys97 and Asn72) and 3 out of the 4 hotspots in the 1 m9 protein (Figure 3). Since this complex is F86A mutant of the Dnase-1 m9 complex the Phe86 of DNase and its interacting partner Tyr55 of 1 m9 were not picked up by our program.
- Subtilisin BPN’ – Chymotrypsin inhibitor 2
  Chymotrypsin inhibitor 2 (CI2) inhibits the serine protease subtilisin by binding to its active site (PDBID: ITM1). A series of mutants have been found to affect the binding of chymotrypsin inhibitor to subtilisin. It has been shown that the network of hydrogen bonds and electrostatic interactions connecting the CI2 binding loop to the protein core provides structural integrity and conformational stability relevant both for binding affinity and for control of inhibitor religation. The H-bond between Thr-58 and Glu-60 bridges the cleavage site, while the interactions between Gly-83, Arg-65, and Glu-60 tie the leaving group R‘-peptide tightly to the protein core, assisting in leaving group retention and accelerating the religation reaction. It has also been shown that mutation of Arg-62, a peripheral participant in the H-bonding network, has comparatively little effect on hydrolysis or inhibition, while mutation of Arg-67 has an intermediate effect [56]. Similarly the importance of Met-59 and Tyr-61 has been described in [57]. Among the above described hotspot residues our program was able to predict five out of eight reported residues (Figure 4).

## Conclusion

Protein-protein interaction hotspot refers to a residue or cluster of residues that makes a major contribution to the binding free energy of protein-protein complexes, as determined by alanine scanning mutagenesis. These residues serve as important targets in the field of pharmaceutical industry for the impedance of certain protein-protein complexes. A number of recent studies have been successful in developing (drug-like) small molecules that bind at hotspots and inhibit complex formation. Experimental identification of hotspot residues is however expensive and time-consuming, and computational methods can thus be helpful in suggesting residues for possible experimentation. In this paper, we describe a novel algorithm which performs better than the existing methods for the identification of hotspot residues validated using previously established experimental data. The method records the highest accuracy available so far for the prediction of hotspots at protein-protein interaction sites.

## Electronic supplementary material

Additional file 1: **Hotspot and non-hotspot residues from the Alanine scanning database used as the training set.** (XLSX 18 KB)

Additional file 2: **Hotspot and non-hotspot residues from the BID database used as the independent test set.** (XLSX 14 KB)

Additional file 3: **Weight ranges and their respective increments used during optimization process.** (XLSX 11 KB)

Additional file 4: **Details on optimization of the weights.** (DOCX 12 KB)

Additional file 5: **Availability of homologues at different sequence identity threshold for some PDB entries.** (XLSX 11 KB)

Additional file 6: **Threshold values for each scoring scheme and corresponding “true positive rate” and “false positive rate”.** (XLSX 24 KB)

## Abbreviations

ECMIS: Energetic, conservation, mass index and spatial clustering
ROC: Receiver operating characteristics plot
ASEdb: Alanine scanning energetic database
BID: Binding interface database
PDB: Protein databank.

## References

[1] Clackson T, Wells JA (1995). A hot spot of binding energy in a hormone-receptor interface. Science.

[2] Bogan AA, Thorn KS (1998). Anatomy of hot spots in protein interfaces. J Mol Biol.

[3] Lise S, Archambeau C, Pontil M, Jones DT (2009). Prediction of hot spot residues at protein–protein interfaces by combining machine learning and energy-based methods. BMC Bioinformatics.

[4] Keskin O, Ma B, Nussinov R (2005). Hot regions in protein–protein interactions: the organization and contribution of structurally conserved hot spot residues. J Mol Biol.

[5] Moreira IS, Fernandes PA, Ramos MJ (2007). Hot spots—a review of the protein–protein interface determinant amino-acid residues. Proteins.

[6] Thorn KS, Bogan AA (2001). ASEdb: a database of alanine mutations and their effects on the free energy of binding in protein interactions. Bioinformatics.

[7] Fischer TB, Arunachalam KV, Bailey D, Mangual V, Bakhru S, Russo R, Huang D, Paczkowski M, Lalchandani V, Ramachandra C, Ellison B, Galer S, Shapley J, Fuentes E, Tsai J (2003). The binding interface database (BID): a compilation of amino acid hot spots in protein interfaces. Bioinformatics.

[8] Burgoyne N, Jackson R (2006). Predicting protein interaction sites: binding hotspots in protein-protein and protein-ligand interfaces. Bioinformatics.

[9] Guharoy M, Chakrabarti P (2005). Conservation and relative importance of residues across protein-protein interfaces. Proc Natl Acad Sci.

[10] Li J, Liu Q (2009). ‘Double water exclusion’: a hypothesis refining the O-ring theory for the hot spots at protein interfaces. Bioinformatics.

[11] Liu Q, Li J (2010). Propensity vectors of low-ASA residue pairs in the distinction of protein interactions. Proteins.

[12] Grishin NV, Phillips MA (1994). The subunit interfaces of oligomeric enzymes are conserved to a similar extent to the overall protein sequences. Protein Sci.

[13] Valdar WS, Thornton JM (2001). Protein-protein interfaces: analysis of amino acid conservation in homodimers. Proteins.

[14] Fraser HB, Hirsh AE, Steinmetz LM, Scharfe C, Feldman MW (2002). Evolutionary rate in the protein interaction network. Science.

[15] Caffrey DR, Somaroo S, Hughes JD, Mintseris J, Huang HS (2004). Are protein-protein interfaces more conserved in sequence than the rest of the protein surface?. Protein Sci.

[16] Panchenko AR, Kondrashov F, Bryant S (2004). Prediction of functional sites by analysis of sequence and structure conservation. Protein Sci.

[17] Yogurtcu ON, Erdemli SB, Nussinov R, Turkay M, Keskin O (2008). Restricted mobility of conserved residues in protein-protein interfaces in molecular simulations. Biophys J.

[18] Kim J, Mao J, Gunner MR (2005). Are acidic and basic groups in buried proteins predicted to be ionized?. J Mol Biol.

[19] Glaser F, Pupko T, Paz I, Bell RE, Bechor-Shental D, Martz E, Ben-Tal N (2003). ConSurf: identification of functional regions in proteins by surface-mapping of phylogenetic information. Bioinformatics.

[20] Wells JA (1991). Systematic mutational analyses of protein-protein interfaces. Methods Enzymol.

[21] Jin L, Wells JA (1994). Dissecting the energetics of an antibody-antigen interface by alanine shaving and molecular grafting. Protein Sci.

[22] Kortemme T, Baker D (2002). A simple physical model for binding energy hot spots in protein-protein complexes. Proc Natl Acad Sci U S A.

[23] Guerois R, Nielsen JE, Serrano L (2002). Predicting changes in the stability of proteins and protein complexes: a study of more than 1000 mutations. J Mol Biol.

[24] Darnell SJ, LeGault L, Mitchell JC (2008). KFC Server: interactive forecasting of protein interaction hot spots. Nucleic Acids Res.

[25] Cho KI, Kim D, Lee D (2009). A feature-based approach to modelling protein–protein interaction hot spots. Nucleic Acids Res.

[26] Tuncbag N, Keskin O, Gursoy A (2010). HotPoint: hot spot prediction server for protein interfaces. Nucleic Acids Res.

[27] Zhu X, Mitchell JC (2011). KFC2: a knowledge-based hot spot prediction method based on interface solvation, atomic density, and plasticity features. Proteins.

[28] Landon MR, Lancia DR, Yu J, Thiel SC, Vajda S (2007). Identification of hot spots within druggable binding regions by computational solvent mapping of proteins. J Med Chem.

[29] Guney E, Tuncbag N, Keskin O, Gursoy A (2008). HotSprint: database of computational hot spots in protein interfaces. Nucleic Acids Res.

[30] Tuncbag N, Gursoy A, Keskin O (2009). Identification of computational hot spots in protein interfaces: combining solvent accessibility and inter-residue potentials improves the accuracy. Bioinformatics.

[31] Li L, Zhao B, Cui Z, Gan J, Sakharkar MK, Kangueane P (2006). Identification of hot spot residues at protein-protein interface. Bioinformation.

[32] Li X, Keskin O, Ma B, Nussinov R, Liang J (2004). Protein-protein interactions: hot spots and structurally conserved residues often locate in complemented pockets that pre-organized in the unbound states: implications for docking. J Mol Biol.

[33] Hu Z, Ma B, Wolfson H, Nussinov R (2000). Conservation of polar residues as hot spots at protein interfaces. Proteins.

[34] Ma B, Elkayam T, Wolfson H, Nussinov R (2003). Protein-protein interactions: structurally conserved residues distinguish between binding sites and exposed protein surfaces. Proc Natl Acad Sci U S A.

[35] Ma B, Nussinov R (2007). Trp/Met/Phe hot spots in protein-protein interactions: potential targets in drug design. Curr Top Med Chem.

[36] Ofran Y, Rost B (2007). Protein-protein interaction hotspots carved into sequences. PLoS Comput Biol.

[37] Hsu CM, Chen CY, Liu BJ, Huang CC, Laio MH, Lin CC, Wu TL (2007). Identification of hot regions in protein-protein interactions by sequential pattern mining. BMC Bioinformatics.

[38] DeLano WL (2002). Unraveling hot spots in binding interfaces: progress and challenges. Curr Opin Struct Biol.

[39] Diller DJ, Humblet C, Zhang X, Westerhoff LM (2010). Computational alanine scanning with linear scaling semiempirical quantum mechanical methods. Proteins.

[40] Massova I, Kollman PA (1999). Computational alanine scanning to probe protein-protein interactions: a novel approach to evaluate binding free energies. J Am Chem Soc.

[41] Assi SA, Tanaka T, Rabbitts TH, Fernandez-Fuentes N (2010). PCRPi: presaging critical residues in protein interfaces, a new computational tool to chart hot spots in protein interfaces. Nucleic Acids Res.

[42] Darnell SJ, Page D, Mitchell JC (2007). An automated decision-tree approach to predicting protein interaction hot spots. Proteins.

[43] Wang L, Liu ZP, Zhang XS, Chen L (2012). Prediction of hot spots in protein interfaces using a random forest model with hybrid features. Protein Eng Des Sel.

[44] Grosdidier S, Fernandez-Recio J (2008). Identification of hot spot residues in protein–protein interactions by computational docking. BMC Bioinformatics.

[45] Gao Y, Wang R, Lai L (2004). Structure-based method for analyzing protein-protein interfaces. J Mol Model.

[46] Gonzalez-Ruiz D, Gohlke H (2006). Targeting protein-protein interactions with small molecules: challenges and perspectives for computational binding epitope detection and ligand finding. Curr Med Chem.

[47] Xia JF, Zhao XM, Song J, Huang DS (2010). APIS: accurate prediction of hot spots in protein interfaces by combining protrusion index with solvent accessibility. BMC Bioinformatics.

[48] Sukhwal A, Sowdhamini R (2013). Oligomerisation status and evolutionary conservation of interface of protein structural domain superfamilies. Mol BioSyst.

[49] Altschul SF, Madden TL, Schäffer AA, Zhang J, Zhang Z, Miller W, Lipman DJ (1997). Gapped BLAST and PSI-BLAST: a new generation of protein database search programs. Nucleic Acids Res.

[50] Li W, Godzik A (2006). Cd-hit: a fast program for clustering and comparing large sets of protein or nucleotide sequences. Bioinformatics.

[51] Larkin MA, Blackshields G, Brown NP, Chenna R, McGettigan PA, McWilliam H, Valentin F, Wallace IM, Wilm A, Lopez R, Thompson JD, Gibson TJ, Higgins DG (2007). ClustalW and ClustalX version 2. Bioinformatics.

[52] Johnson MS, Overington JP, Blundell TL (1993). A structural basis for sequence comparisons: an evaluation of scoring methodologies. J Mol Biol.

[53] Pugalenthi G, Suganthan PN, Sowdhamini R, Chakrabarti S (2007). SMotif: a server for structural motifs in proteins. Bioinformatics.

[54] Wallis R, Leung KY, Osborne MJ, James R, Moore GR, Kleanthous C (1998). Specificity in protein–protein recognition: conserved Im9 residues are the major determinants of stability in the colicin E9 dnase Im9 complex. Biochemistry.

[55] Curtis MD, James R (1991). Investigation of the specificity of the interaction between colicin E9 and its immunity protein by site-directed mutagenesis. Mol Microbiol.

[56] Radisky ES, Lu CJK, Kwan G, Koshland DE (2005). Role of the intermolecular hydrogen bond network in the inhibitory power of chymotrypsin inhibitor 2. Biochemistry.

[57] Radisky ES, Kwan G, Lu CJK, Koshland DE (2004). Binding, proteolytic, and crystallographic analyses of mutations at the protease − inhibitor interface of the subtilisin BPN‘/chymotrypsin inhibitor 2 complex. Biochemistry.

