# Supplementary material for: ECMIS: computational approach for the identification of hotspots at protein-protein interfaces
Source: BMC Bioinformatics. 2014 Sep 16;15(1):303. doi: 10.1186/1471-2105-15-303 (PMC4177600; doi:10.1186/1471-2105-15-303)
Supplement: Supplementary file 4 — Additional file 4: Details on optimization of the weights. (DOCX 12 KB) [file 12859_2014_6585_MOESM4_ESM.docx]

**Details on optimization of the weights:**

To determine hotspot residues in the protein complex, several features were used. The importance of each feature varies in determining the hotspot residues. Hence it is important to find out appropriate weights for each feature according to their importance in identifying the hotspot residues. To decide optimum weights we tried all different combinations of parameters (Supplementary Table 3) for carefully chosen range and increment. Each combination of parameters was evaluated using training dataset. The combination of parameters which yielded maximum accuracy was chosen as final combination of weights for the ECMIS algorithm.

The minimum and maximum values of weights for each energy component were decided after manual observation of score of each energy component and its experimentally known importance.

*E.g.*: Van der Waals interactions which also include hydrophobic interactions are very smaller in terms of magnitude of the energy contributed. In some protein complexes like the complex of cytochrome *f* and plastocyanin hydrophobic residues are very critical for protein-complex formation [60]. Hence such these interactions should be represented with appropriate score. Hence to avoid under-representation of Van der Waals interactions the maximum value of its respective weight used was 15.
